# Supplementary material for: Boron triggers grain boundary structural transformation in steel
Source: Nat Commun. 2025 Jul 28;16:6927. doi: 10.1038/s41467-025-62264-1 (PMC12304139; doi:10.1038/s41467-025-62264-1)
Supplement: Supplementary file 1 — Supplementary Information [file 41467_2025_62264_MOESM1_ESM.pdf]

Supplementary Information for Boron Triggers Grain Boundary Structural Transformation in Steel

Xuyang Zhou[1*†], Sourabh Kumar[2†], Siyuan Zhang[1], Xinren Chen[1], Baptiste Gault[1,3], Gerhard Dehm[1], Tilmann Hickel[1,2*] and Dierk Raabe[1*]

[1]Max-Planck-Institut for Sustainable Materials, Max-Planck-Straße 1, Düsseldorf, 40237, Germany.
[2]Federal Institute for Materials Research and Testing (BAM), Richard-Willstätter-Straße 11, Berlin, 12489, Germany.
[3]Department of Materials, Imperial College London, London, SW7 2AZ, UK.

*Corresponding author(s). E-mail(s): x.zhou@mpie.de; tilmann.hickel@bam.de; raabe@mpie.de;
†These authors contributed equally to this work.

This PDF file includes:

Supplementary Figure 1 to Figure 20

1 Supplemental Figures

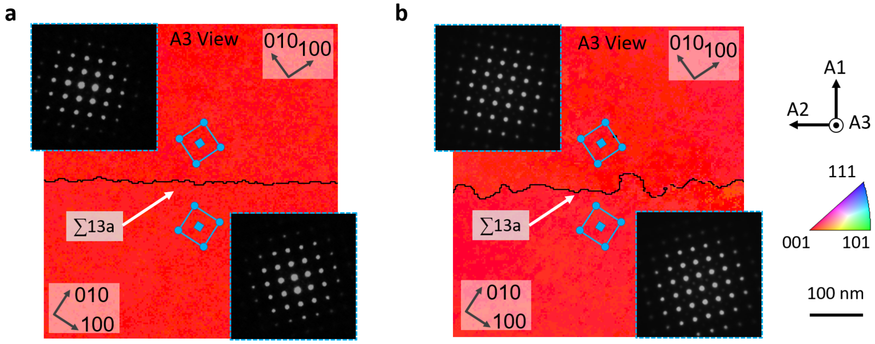

Supplementary Figure 1: Nanoscale orientation mapping of the BCC iron Σ13[001] GBs: **a** Reconstruction of the pristine GB, and **b** reconstruction of the boron-alloyed GB, both derived from 4DSTEM datasets. The orientation maps correspond to the A3 view using sample coordinates A1-A3. Here, A3 is the direction of the thin film growth. Superimposed images show averaged diffraction patterns from top and bottom grains, with square patterns indicating grain orientations. Coordination systems for upper and lower grains are included for comparison.

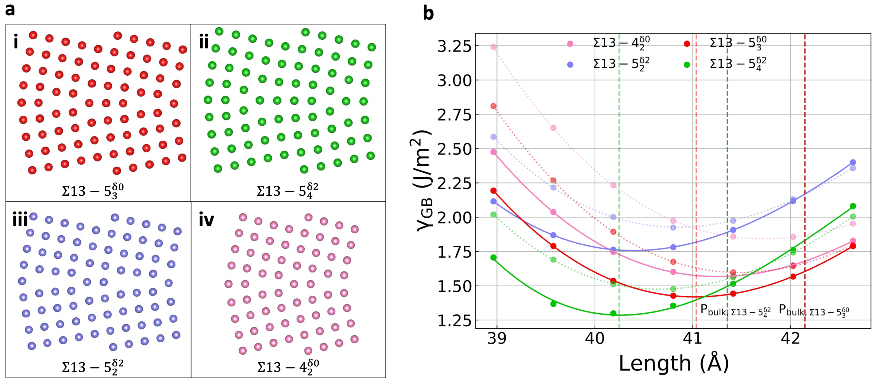

Supplementary Figure 2: Modeling of the pristine iron Σ13[001] **GB atomic configurations**: **a** Σ13[001] GB atomic configurations for the BCC iron showing potential defects at the GB interfaces: **i** The GB atomic configuration without any defects, represented as $\Sigma 13 - 5_3^{\delta_0}$ (Flat B-type). In this notation, "5" before the subscript indicates the apparent smallest repeating unit cell along the GB as observed in imaging. The subscript "3" denotes the number of atomic sequences at the GB interface, and δ_0 signifies the absence of defects. **ii - iv** Other possible GB atomic configurations with varying defect counts and atomic sequences at the GB interface are shown as follows: **ii** $\Sigma 13 - 5_4^{\delta_2}$ (Flat A-type), where "5" represents the repeating unit cell and "4" the number of atomic sequences, with defects involving two missing iron atoms indicated by δ_2; **iii** $\Sigma 13 - 5_2^{\delta_2}$, similar to **ii** but with two atomic sequences and defects from two displaced iron atoms. **iv** $\Sigma 13 - 4_2^{\delta_0}$, where "4" is the repeating unit and "2" the number of atomic sequences, contains no defects. **b** GB energy as a function of lattice parameters and the length normal to the GB plane for different Σ13[001] GB atomic configurations. Here, a color-coding scheme is used to distinguish between the different GB atomic configurations. The solid and dashed lines represent lattice parameters of 2.870 Å and 2.832 Å, respectively.

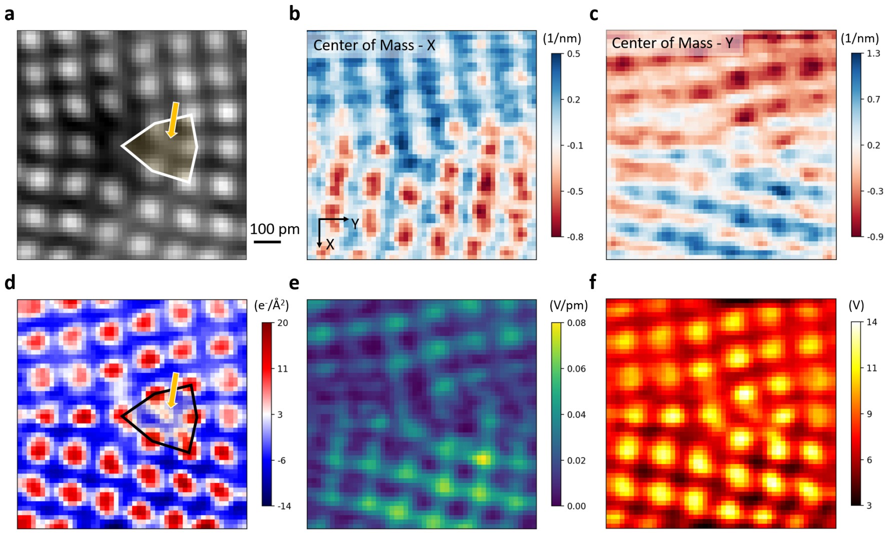

Supplementary Figure 3: Experimental DPC-4DSTEM reconstruction for the pristine iron $\Sigma 13[001]$ GB to image the light carbon atomic column (one site in the imaged area). **a** Reconstructed virtual dark-field image. Change of the center of mass of the transmitted beam in the **b** X and **c** Y directions. **d** Charge-density map. **e** Electric field vector map. **f** Projected electrostatic potential map. The scanning step size used in this experiment is 18 pm.

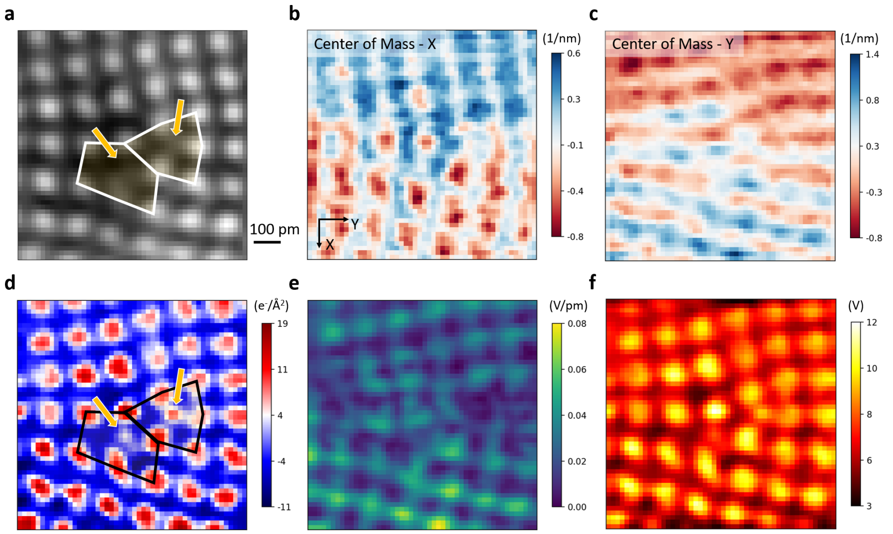

Supplementary Figure 4: Experimental DPC-4DSTEM reconstruction for the pristine iron $\Sigma 13[001]$ GB to image light carbon atomic columns (two sites in the imaged area). **a** Reconstructed virtual dark-field image. Change of the center of mass of the transmitted beam in the **b** X and **c** Y directions. **d** Charge-density map. **e** Electric field vector map. **f** Projected electrostatic potential map. The scanning step size used in this experiment is 18 pm.

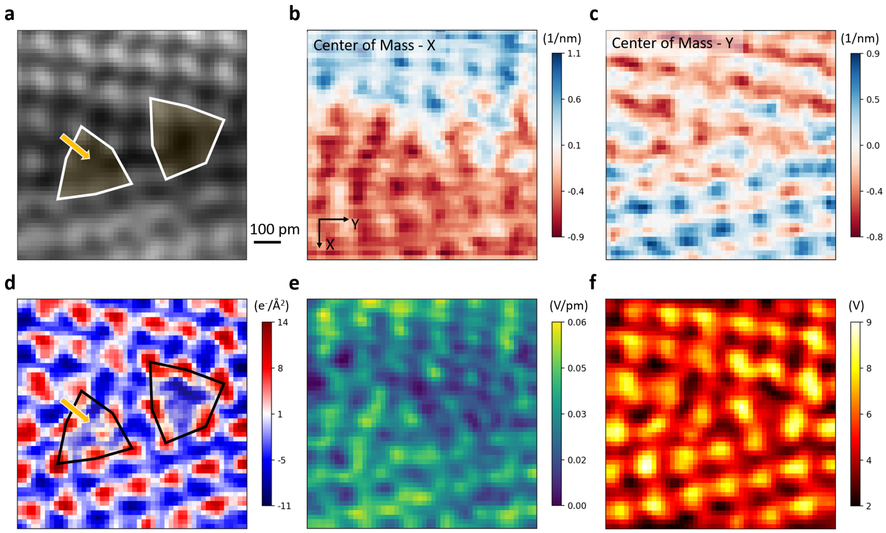

Supplementary Figure 5: Experimental DPC-4DSTEM reconstruction for the boron-alloyed iron Σ13[001] GB to image the light boron/carbon atomic column (one site in the imaged area). a Reconstructed virtual dark-field image. Change of the center of mass of the transmitted beam in the **b** X and **c** Y directions. **d** Charge-density map. **e** Electric field vector map. **f** Projected electrostatic potential map. The scanning step size used in this experiment is 18 pm.

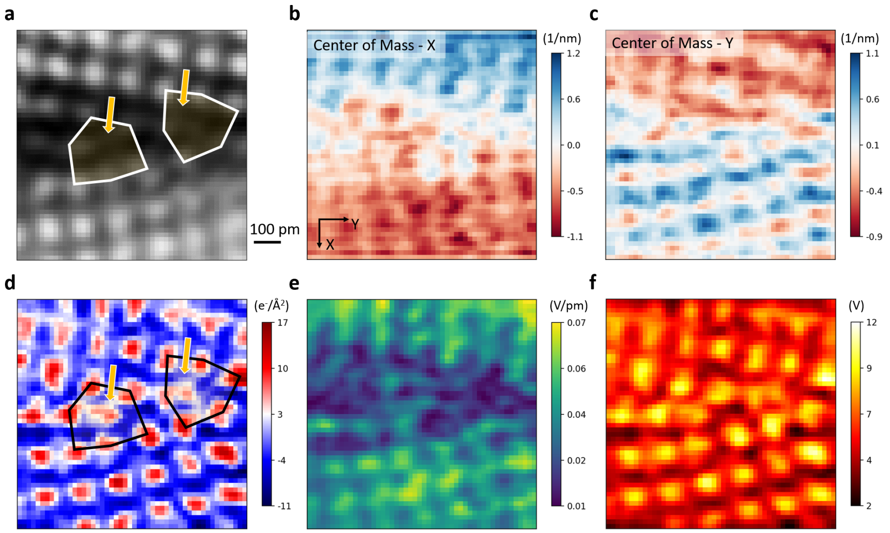

Supplementary Figure 6: Experimental DPC-4DSTEM reconstruction for the boron-alloyed iron $\Sigma 13[001]$ GB to image the light boron/carbon atomic columns (two sites in the imaged area). a Reconstructed virtual dark-field image. Change of the center of mass of the transmitted beam in the **b** X and **c** Y directions. **d** Charge-density map. **e** Electric field vector map. **f** Projected electrostatic potential map. The scanning step size used in this experiment is 18 pm.

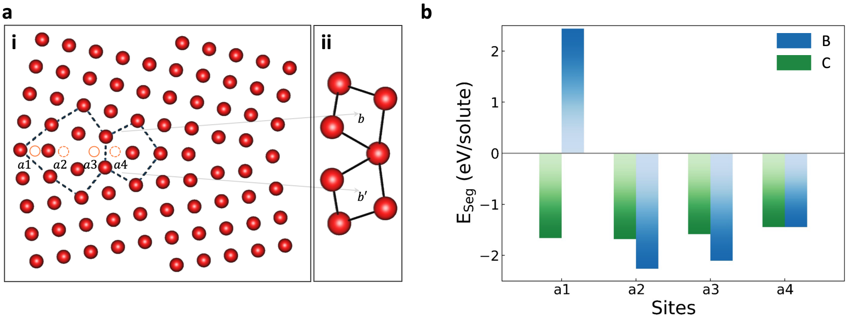

Supplementary Figure 7: Solute segregation (boron and carbon) at the BCC iron $\Sigma 13 - 5_3^{\delta 0}$ GB (Flat B-type): **a i** Possible sites for the solute segregation (boron and carbon) at the GB interface. The red sphere with dashed lines is in the same plane as the black dashed lines. **ii** Nearest pathway or voids for the diffusion of solutes away from GB based on Voronoi analysis. **b** The segregation energy are compared for the boron and carbon solute atom at the GB for the different interstitial sites.

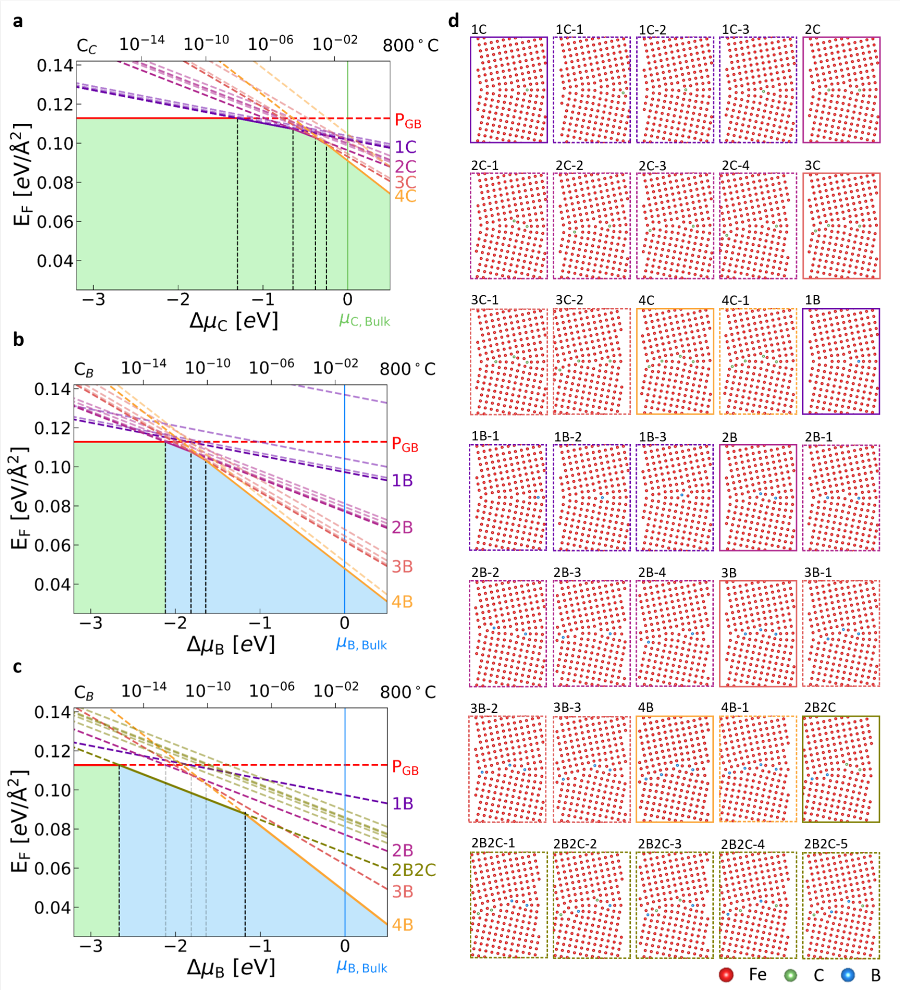

Supplementary Figure 8: DPDs for the BCC iron $\Sigma 13[001]$ GB with additional lines representing metastable atomic configurations: DPDs of Figs. 3a-c, where the energies of the metastable GB atomic configurations have been added as lighter dashed lines. **a & b** The formation energies for GB atomic configurations are plotted in reference to $\Delta\mu_{\mathrm{C}}$ and $\Delta\mu_{\mathrm{B}}$. Each line represents an atomic configuration with increased boron or carbon atoms at the $\Sigma 13[001]$ GB. **c** The formation energies for atomic configurations with equal boron and carbon atoms (2B2C) at the GB, are plotted with $\Delta\mu_{\mathrm{B}}$ at fixed μ_{C}. **d** The DFT-relaxed GB atomic configurations for both stable (solid frames) and metastable (dashed frames). The colors of the frames are consistent with the lines in **a - c**. The numbers following the coverage designations indicate different types of metastable GB atomic configurations related to each level of solute coverage. For instance, "1-3" after 1C specifies three distinct metastable GB atomic configurations related to the 1C structure.

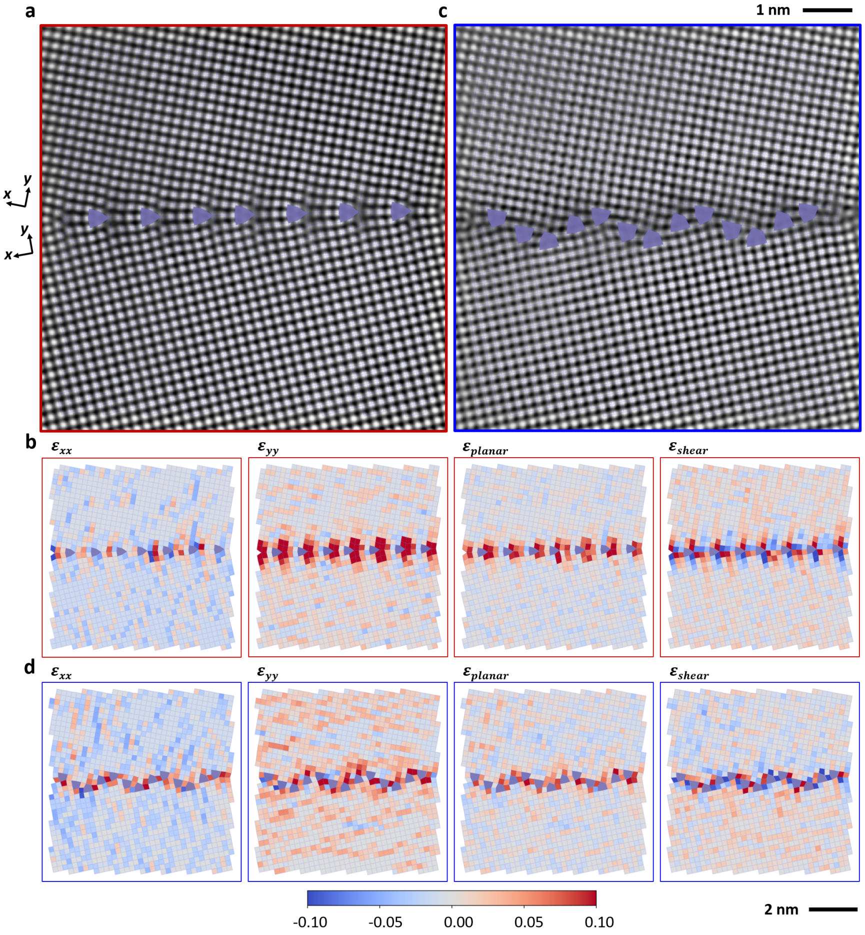

Supplementary Figure 9: Quantification of strain analysis adjacent to the GB plane a & b High-resolution HAADF-STEM images of the BCC iron Σ13[001] GB: **a** for the pristine GB and **b** for the boron-alloyed GB, which correspond to the images presented in Figs. 1 a & b, respectively. Overlaid on these images are a light blue grid indicating regions of maximal symmetry identified through automated registration, and purple-colored hexagonal shapes representing the trigonal prisms at the GBs. **c & d** Quantification of the ϵ_{xx} and ϵ_{yy} strains (along the $\langle 110 \rangle$ crystalline direction) and the planar and shear strains adjacent to the GB plane from the high-resolution HAADF-STEM images shown in **a & b**: **c** for the pristine GB and **d** for the boron-alloyed GB.

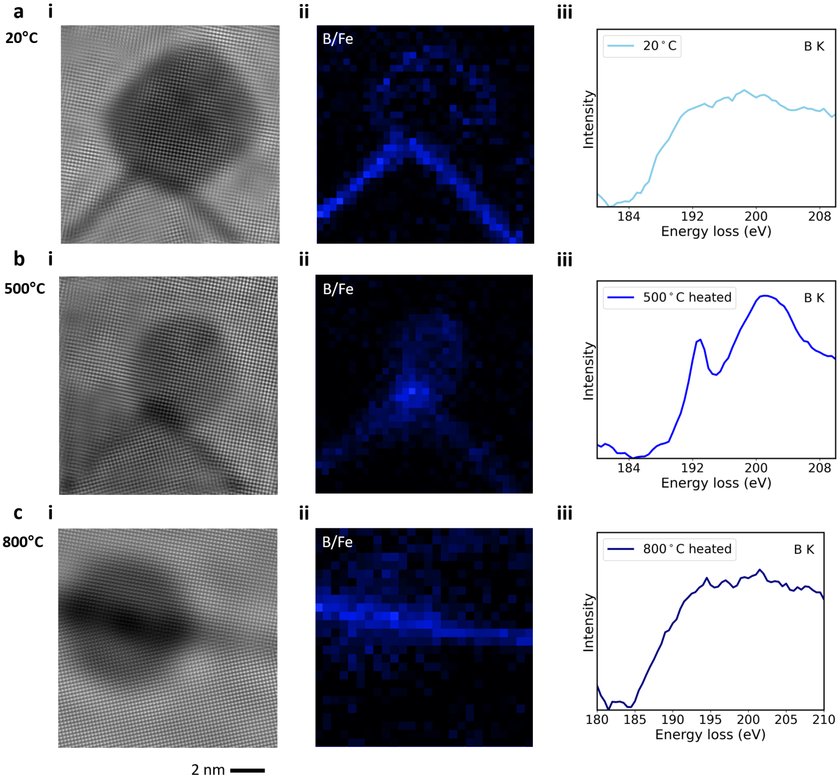

Supplementary Figure 10: EELS mapping reveals the chemistry at the iron $\Sigma 13[001]$ with boron segregation at elevated temperatures: EELS data were collected at **a** 20 °C, **b** after being heat-treated at 500 °C, and **c** after being heat-treated at 800 °C. The heating profiles are referred to Fig. 4e. In each sub-figure, **i** is the HAADF-STEM image, **ii** shows the boron to iron signal, and **iii** is the corresponding spectrum.

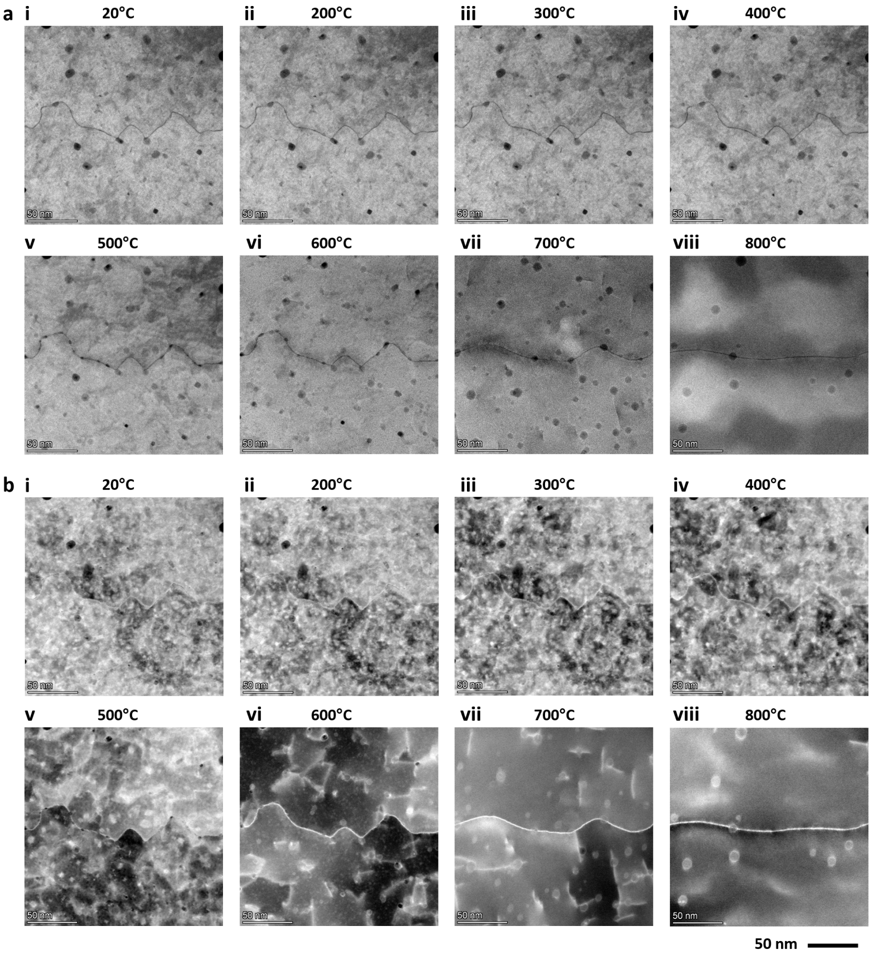

Supplementary Figure 11: Overview of the iron Σ13[001] GB morphology evolution at different temperatures of the boron-alloyed sample: **a** HAADF-STEM images and **b** DF4-STEM images showing the evolution of the GB morphology at temperatures ranging from **i** 20 °C to **viii** 800 °C for the sample with boron segregation.

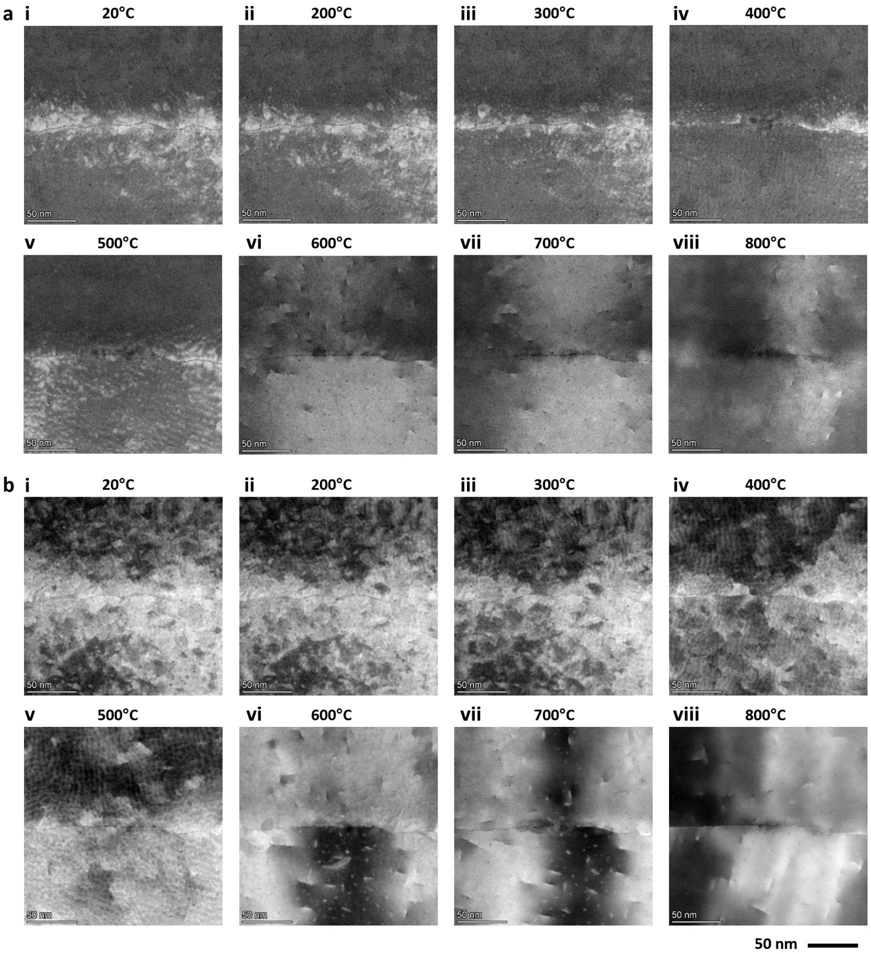

Supplementary Figure 12: Evolution of the iron Σ13[001] GB morphology in pristine sample at different temperatures: HAADF-STEM images illustrating the morphological changes in the GBs of the pristine sample at temperatures ranging from **i** 20 °C to **viii** 800 °C. **a** HAADF-STEM images and **b** dark-field four (DF4)-STEM images are shown.

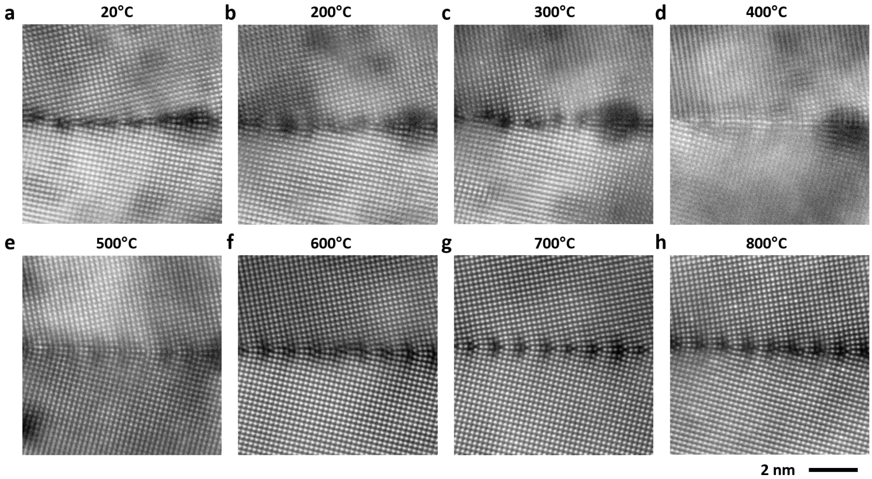

Supplementary Figure 13: The atomic structure evolution of the BCC iron $\Sigma 13[001]$ GB with a GB plane close to the symmetric $\{320\}$ plane in the pristine sample: Raw high-resolution HAADF-STEM images illustrating the changes in atomic structure at the GBs of the pristine sample over a temperature range from **a** 20 °C to **h** 800 °C. Here, **g** is the raw high-solution HAADF-STEM image of Fig. 1a and Supplementary Fig. 9a

.

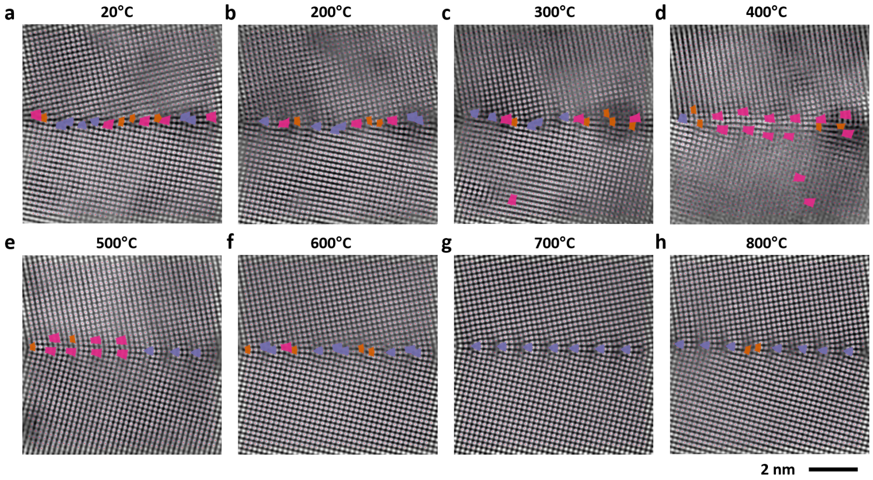

Supplementary Figure 14: The atomic structure evolution of the BCC iron $\Sigma\mathbf{13}[001]$ GB with a GB plane close to the symmetric $\{320\}$ plane in the pristine sample: High-resolution HAADF-STEM images illustrating the changes in atomic structure at the GBs of the pristine sample over a temperature range from **a** 20 °C to **h** 800 °C. Overlaying these images are red markers pinpointing atomic column locations, a light blue grid indicating regions of maximal symmetry identified through automated registration, and variably colored shapes highlighting deviations from square symmetry: orange for pentagonal, purple for hexagonal, and pink for heptagonal shapes.

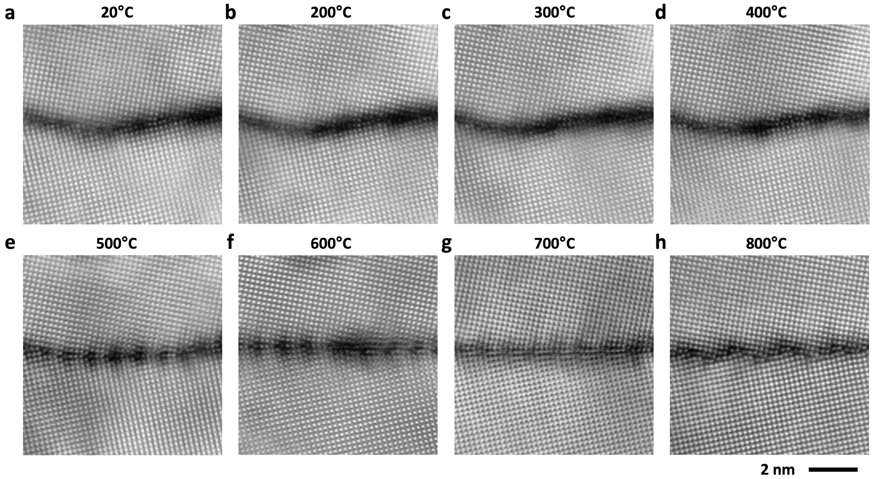

Supplementary Figure 15: The atomic structure evolution of the BCC iron $\Sigma 13[001]$ GB with a GB plane close to the symmetric $\{320\}$ plane in the boron-alloyed sample: Raw high-resolution HAADF-STEM images illustrating the changes in atomic structure at the GBs of the pristine sample over a temperature range from **a** 20 °C to **h** 800 °C. Here, **h** is the raw high-solution HAADF-STEM image of Fig. 1b and Supplementary Fig. 9b

.

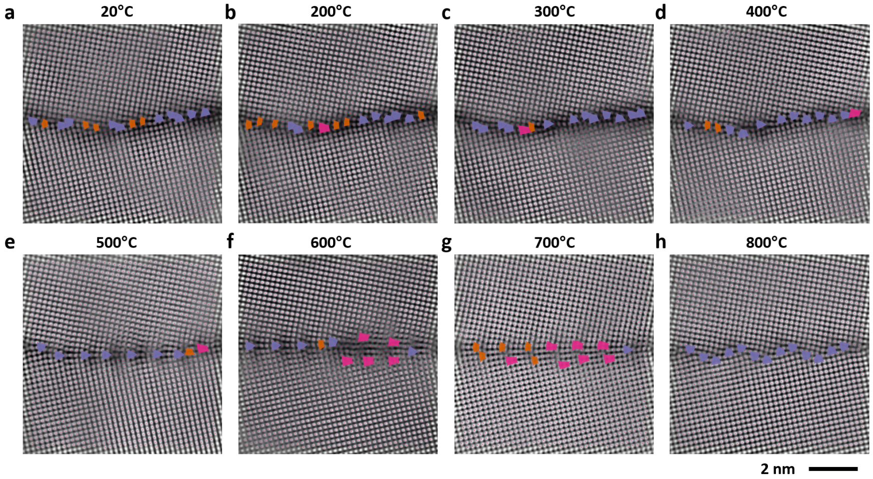

Supplementary Figure 16: The atomic structure evolution of the BCC iron Σ13[001] GB with a GB plane close to the symmetric {320} plane in the boron-alloyed sample: High-resolution HAADF-STEM images illustrating the changes in atomic structure at the GBs of the boron-alloyed sample over a temperature range from **a** 20 °C to **h** 800 °C. Overlaying these images are red markers pinpointing atomic column locations, a light blue grid indicating regions of maximal symmetry identified through automated registration, and variably colored shapes highlighting deviations from square symmetry: orange for pentagonal, purple for hexagonal, and pink for heptagonal shapes.

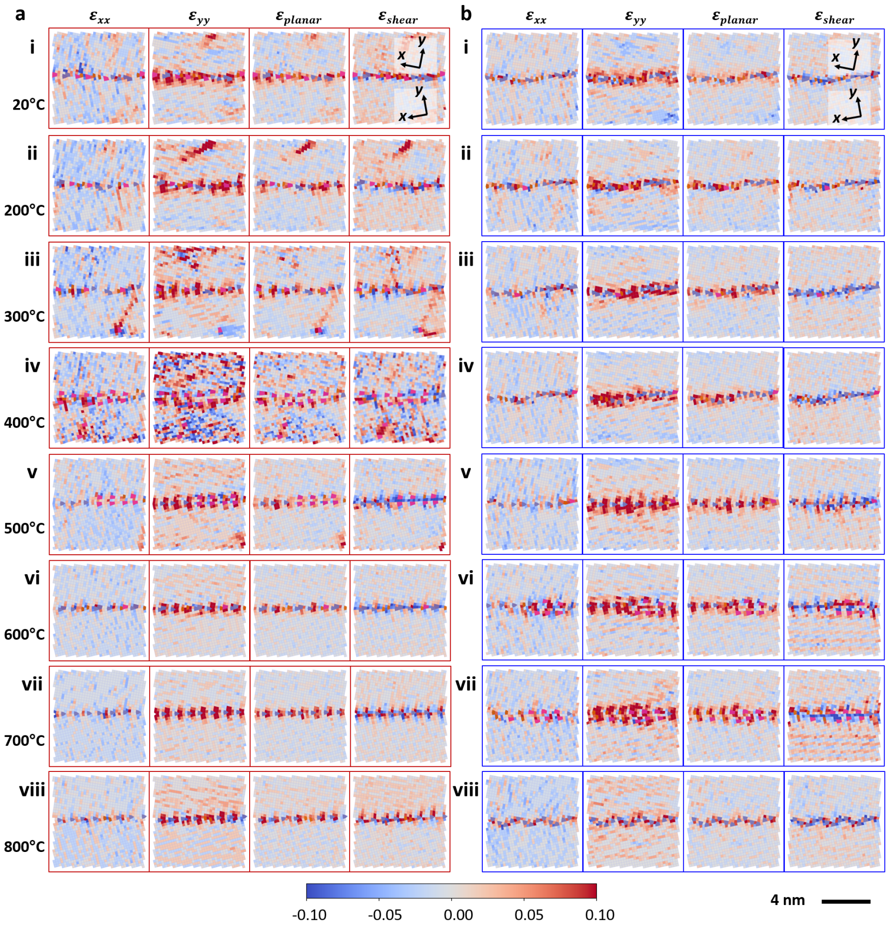

Supplementary Figure 17: Quantification of strain evolution adjacent to the GB plane from the high-resolution HAADF-STEM images of the iron Σ13[001] GBs: **a** for the pristine GB, **b** for the boron-alloyed GB. In each sub-figure, **i-viii** indicate the temperature range from **i** 20 °C to **viii** 800 °C. From left to right, the figures show the quantification of the ϵ_{xx} and ϵ_{yy} strains (along the ⟨110⟩ crystalline direction) and the planar and shear strains adjacent to the GB plane from the high-resolution HAADF-STEM images shown in Supplementary Figs. 14 and 16.

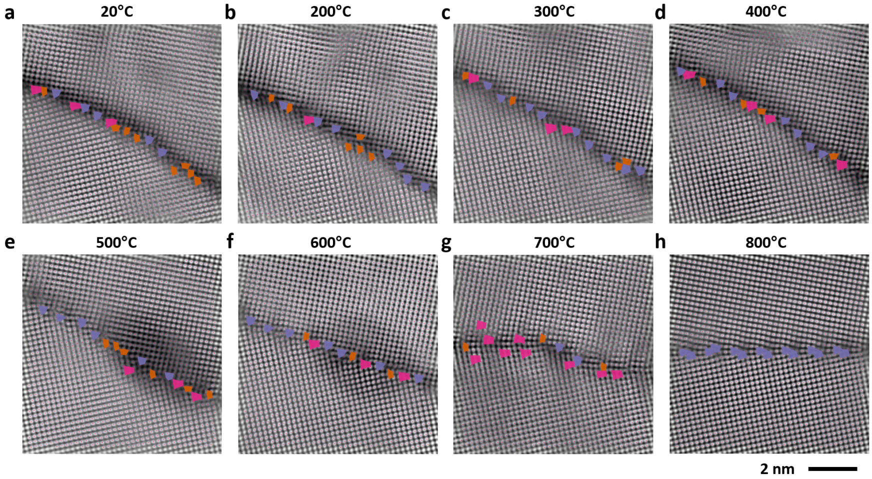

Supplementary Figure 18: The atomic structure evolution of the iron $\Sigma 13[001]$ in the boron-alloyed sample, starting with an initial GB plane at $20\,^{\circ}\mathrm{C}$ oriented $\approx 30^{\circ}$ away from the symmetric $\{320\}$ plane: High-resolution HAADF-STEM images illustrating the changes in atomic structure at the GBs of the boron-alloyed sample over a temperature range from **a** $20\,^{\circ}\mathrm{C}$ to **h** $800\,^{\circ}\mathrm{C}$. Overlaying these images are red markers pinpointing atomic column locations, a light blue grid indicating regions of maximal symmetry identified through automated registration, and variably colored shapes highlighting deviations from square symmetry: orange for pentagonal, purple for hexagonal, and pink for heptagonal shapes.

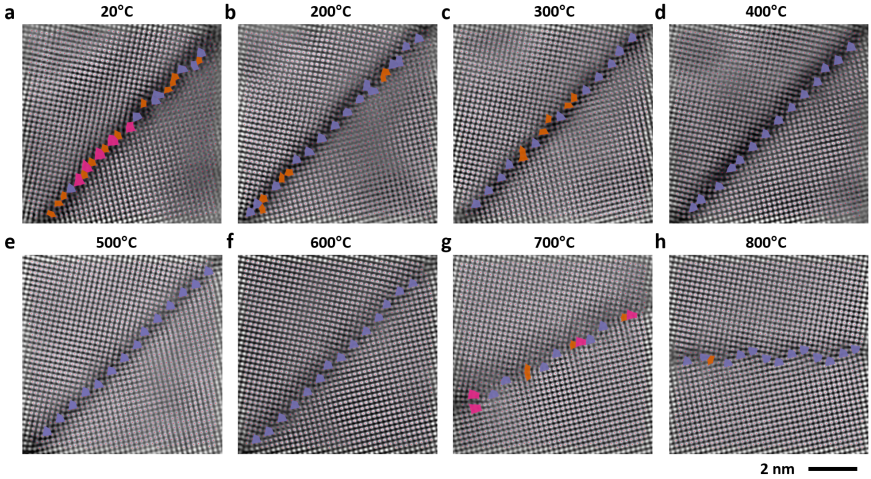

Supplementary Figure 19: The atomic structure evolution of the iron $\Sigma 13[001]$ in the boron-alloyed sample, starting with an initial GB plane at $20\,^{\circ}\mathrm{C}$ oriented $\approx 45^{\circ}$ away from the symmetric $\{320\}$ plane: High-resolution HAADF-STEM images illustrating the changes in atomic structure at the GBs of the boron-alloyed sample over a temperature range from **a** $20\,^{\circ}\mathrm{C}$ to **h** $800\,^{\circ}\mathrm{C}$. Overlaying these images are red markers pinpointing atomic column locations, a light blue grid indicating regions of maximal symmetry identified through automated registration, and variably colored shapes highlighting deviations from square symmetry: orange for pentagonal, purple for hexagonal, and pink for heptagonal shapes.

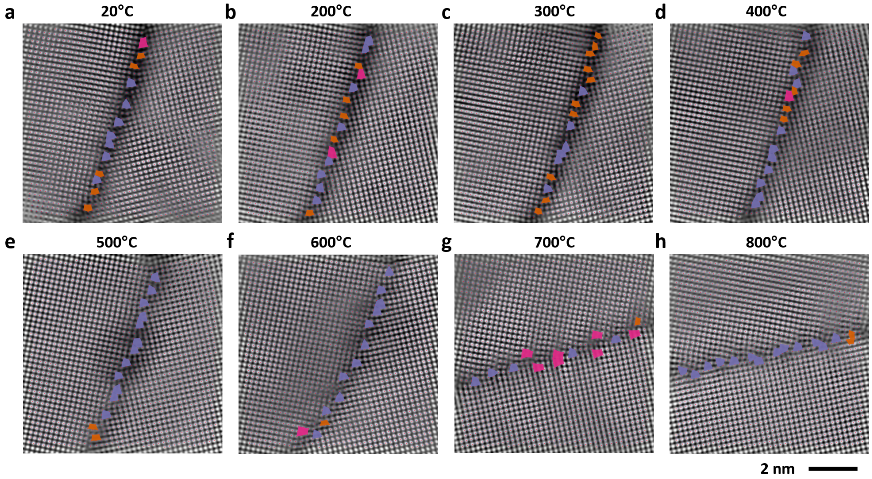

Supplementary Figure 20: The atomic structure evolution of the iron Σ13[001] in the boron-alloyed sample, starting with an initial GB plane at 20 °C oriented ≈ 75° away from the symmetric {320} plane: High-resolution HAADF-STEM images illustrating the changes in atomic structure at the GBs of the boron-alloyed sample over a temperature range from **a** 20 °C to **h** 800 °C. Overlaying these images are red markers pinpointing atomic column locations, a light blue grid indicating regions of maximal symmetry identified through automated registration, and variably colored shapes highlighting deviations from square symmetry: orange for pentagonal, purple for hexagonal, and pink for heptagonal shapes.
